# Establishment of a Keratin 14/17-Induced Antigen-Specific Psoriasis-like Mouse Model

**DOI:** 10.3390/biology15151277

**Published:** 2026-08-03

**Authors:** Lanying Wang, Yulu Tang, Wenjing Li, Pengcheng Zhu, Wenjing Jia, Hao Liu, Yuanfang Ma, Guimei Wang, Ruiling Liu, Qingguo Ruan, Shijun J. Zheng

**Affiliations:** 1 Joint National Laboratory for Antibody Drug Engineering, School of Synthetic Biology and Pharmaceutical Engineering, Henan University, Kaifeng 475004, China; lan131600@163.com (L.W.); 18238290585@163.com (Y.T.); 15324932155@163.com (W.L.); zpc17550606703@163.com (P.Z.); 16638829374@163.com (W.J.); lhzs17627162007@163.com (H.L.); mayf@henu.edu.cn (Y.M.); 17726067138@163.com (G.W.); 2 School of Basic Medical Sciences, Henan University School of Medicine, Kaifeng 475004, China

**Keywords:** psoriasis, mouse model, keratin 14, keratin 17, autoantigen

## Abstract

Psoriasis is a chronic inflammatory autoimmune skin disease significantly impairing the patients’ quality of life. Keratin (K)14 and K17 are abnormally expressed in psoriasis patients and considered to be potential autoantigens triggering this disease. However, the animal model for psoriasis induced with a specific antigen is yet to be established. Here we show that vaccinating BALB/c and C57BL/6J mice with purified murine recombinant K14 or K17 antigen induced psoriasis-like clinical symptoms in 40 ± 30% of animals, such as erythema and scaling, accompanied by hallmark histopathological changes including significant epidermal hyperplasia and inflammatory cell infiltration in the lesions. Furthermore, the immunized mice produced high levels of specific antibodies against K14/K17 in the blood, along with antigen-specific T-cell responses. These data indicate that immunization of mice with K14 and K17 antigens could cause psoriasis-like symptoms with immunopathological features of psoriasis, providing a novel experimental animal model for investigating the pathogenesis of psoriasis and development of effective therapies.

## Simple Summary

Psoriasis is an autoimmune disease triggered by the immune system primarily attacking host skin. Animal models are required to recapitulate the pathological process of this disease. This study focused on two potential autoantigens found in psoriasis patients, keratin (K)14 and K17, and investigated whether vaccinating mice with these antigens could establish a psoriasis animal model. Our results show that vaccination with K14 or K17 successfully induced psoriasis-like symptoms in mice, including erythema, scales, and typical histopathological changes. This antigen-specific model is capable of mimicking the key features of psoriasis, providing a more precise experimental tool for studying the pathogenesis of the autoimmune disease and for the development of effective therapies with an ultimate goal of curing patients.

## 1. Introduction

Psoriasis is a chronic and recurrent autoimmune skin disease that can affect individuals across different regions, ethnicities and ages, with a global prevalence of approximately 2–3% [1,2]. Its typical clinical manifestations include well-demarcated erythematous plaques or maculopapules covered with silvery-white scales, often accompanied by significant itching, pain, and other symptoms, thereby severely impairing patients’ quality of life and social functioning. The disease is currently incurable, and existing treatment options, including topical therapies, systemic treatments and biologic agents, can only control and delay disease progression. The absence of antigen-induced psoriasis animal models has greatly impeded research into the pathogenesis of psoriasis and the development of curative therapies. Thus, establishment of antigen-induced psoriasis animal models is urgently needed for further investigation into psoriasis.

The pathogenesis of psoriasis involves a complex interplay among genetic susceptibility, immune dysregulation, and environmental triggering factors [3,4,5]. In psoriasis, particularly guttate psoriasis, infection with group A β-hemolytic *Streptococcus* can initiate autoimmune responses through molecular mimicry [6]. Keratin (K)17, primarily expressed in basal keratinocytes of stratified epithelium, has been identified as a target antigen recognized by autoreactive T cells [7,8]. Its “ALEEANxxL” and “GLRRxLD” motifs are cross-reactive epitopes shared with the M protein of group A β-hemolytic *Streptococcus* [7,9]. Keratin 14 (K14) is another type I keratin predominantly expressed in the basal layer of stratified epithelium, playing a crucial role in maintaining epidermal structural integrity and exhibiting aberrant expression in psoriasis [10]. It also contains “ALEEANxxL” and “GLRRxLD” motifs similar to those of the M protein in *Streptococcus*. Both K14 and K17 proteins are highly conserved between humans and mice, suggesting that they may have retained important epitopes throughout evolution. Thus, K14, akin to K17, may also serve as a potential autoantigen involved in psoriasis.

Although current topical, systemic, and biologic therapies could control psoriasis effectively for a certain period of time, the disease relapses following withdrawal of medication. Furthermore, the precise mechanisms underlying disease initiation and relapse remain elusive. Progress in psoriasis research is greatly impeded largely due to the unavailability of antigen-induced psoriasis animal models. Existing mouse models of psoriasis are predominantly drug-induced models, such as inflammation triggered by imiquimod (IMQ) [11], which exhibit acute non-specific inflammatory responses and pathological changes, and fail to recapitulate the autoimmune process initiated by specific autoantigens in human disease [12]. Various cell-based and tissue-engineered psoriasis models have been developed for mechanistic studies, but they lack an intact immune system and cannot model antigen-specific autoimmunity [13]. To further investigate the pathogenesis and therapeutic development of psoriasis, an antigen-induced animal model of the disease is urgently needed.

In this study, murine K14 and K17 proteins were cloned, expressed, purified, and injected into mice to induce psoriasis-like symptoms. It was found that injection of mice with K14 and K17 antigens induced psoriasis-like symptoms associated with high levels of antibodies against the antigens and enhanced responses of K14- and K17-specific T cells, indicating that an antigen-specific psoriasis mouse model has been established in this study, providing a useful animal model for elucidating the pathogenesis of psoriasis and for the development of effective therapies.

## 2. Materials and Methods

### 2.1. Animals

Wild-type female C57BL/6J and BALB/c mice (6–8 weeks old) were obtained from Vital River Laboratory Animal Technology Co., Ltd. (Beijing, China). All animals were housed under specific pathogen-free (SPF) conditions at 22 ± 2 °C with 50 ± 10% relative humidity on a 12 h light/dark cycle and had free access to food and water. All experimental procedures were approved by the Laboratory Animal Ethics Committee of the National Local Joint Engineering Laboratory of Antibody Drug Development at Henan University (approval No. DWLL20250603) and were performed in accordance with its regulations and guidelines.

### 2.2. Construction of Expression Vector

The coding sequences (CDSs) of mouse K14 and K17 were separately ligated into the pGEX-4T-1 vector containing a glutathione S-transferase (GST) tag, or into the pCold vector containing a His-Trigger factor (TF) tag. The primers for gene cloning (GST-K14-F: ctgtattttcagggcatggccacctgcagccgccag, GST-K14-R: tcgacccgggaattcttagttcttggtgcgcagg, GST-K17-F: ctgtattttcagggcatgaccaccaccatccgccag, GST-K17-R: tcgacccgggaattcttaacgggtggtctggtgcacc, TF-K14-F: ctgtattttcagggcatggccacctgcagccgccag, TF-K14-R: gacaagcttgaattcttagttcttggtgcgcagg, TF-K17-F: ctgtattttcagggcatgaccaccaccatccgccag, TF-K17-R: gacaagcttgaattcttaacgggtggtctggtgc) were synthesized at Beijing Tsingke Biotechnology Co., Ltd. (Beijing, China). The CDSs of murine K14 and K17 genes with homology arms were amplified by PCR, purified and recovered, and linearized vectors were obtained by inverse PCR, followed by homologous recombination to generate recombinant products. These were transformed into *Escherichia coli* DH5α competent cells, and single colonies were picked for target fragment amplification and sequencing. Plasmids were extracted using a kit (DP103, Tiangen, Beijing, China), and the recombinant plasmids were designated as pGEX-4T-1-GST, pGEX-4T-1-GST-K14, pGEX-4T-1-GST-K17, as well as pCold-His-TF, pCold-His-TF-K14, and pCold-His-TF-K17. The plasmids were transformed into *Escherichia coli* BL21 competent cells for protein expression.

### 2.3. Purification of K14 and K17 Antigens

Single colonies were picked and inoculated into fresh LB liquid medium containing ampicillin, followed by overnight culture at 37 °C with shaking at 200 rpm. The overnight culture was then transferred into fresh medium at a 1:100 ratio and incubated at 37 °C with shaking at 200 rpm until the OD600 reached 0.6–0.8. Protein expression was induced by adding 0.1 mM IPTG, followed by overnight incubation at 16 °C. After centrifugation at 8000× *g* for 10 min, the bacterial pellets were collected and washed. An appropriate volume of GST binding buffer (50 mM Tris, 150 mM NaCl, 1 mM EDTA, 5 mM DTT, 1% Triton X-100, pH 7.4) or His binding buffer (50 mM Tris, 150 mM NaCl, 13 mM imidazole, 1 mM EDTA, 1% Triton X-100, pH 7.4) was added to resuspend the bacterial pellets. Cells were disrupted using a high-pressure homogenizer at 900 bar for two cycles, followed by centrifugation at 12,000× *g* for 15 min to collect the supernatant. The affinity chromatography column was first equilibrated with 5–10 column volumes of binding buffer at a constant flow rate. The supernatant was then loaded onto the column at a slow flow rate to allow sufficient binding of the target protein to Glutathione Sepharose 4B (17075601, Cytiva, Marlborough, MA, USA) or Ni-TED Agarose Resin 6FF His (20497ES50, Yeasen, Shanghai, China). The column was washed with 2–3 column volumes of binding buffer to remove non-specifically bound proteins. Elution was then performed using GST elution buffer (50 mM Tris, 150 mM NaCl, 50 mM reduced glutathione, 2 mM DTT, pH 8.0) or His elution buffer (50 mM Tris, 150 mM NaCl, 200 mM imidazole, 2 mM DTT, 1 mM EDTA, 1% Triton X-100, pH 7.4), followed by buffer exchange with PBS at 4 °C for >15 h with three buffer changes. The dialyzed proteins were filtered using a 0.22 µm filter. Protein purity was assessed by SDS-PAGE, and protein concentration was determined using the Bicinchoninic Acid (BCA) method. Endotoxin levels were minimized by using endotoxin-free consumables and were quantified by Kinetic Turbidimetric LAL Assay (60401ES06, Yeasen,), with only standard curves exhibiting R^2^ ≥ 0.99 accepted for quantification. In most preparations, endotoxin levels were below 1 EU/mL.

### 2.4. Western Blot

Purified proteins were resolved by 10% SDS-PAGE at 90 V (stacking) and 120 V (resolving). Gels were either stained with Coomassie Brilliant Blue (10–50 μg/lane) or processed for Western blot (0.5–1 μg/lane). For Western blot, proteins were transferred to PVDF membranes, blocked with 5% skim milk, and probed overnight at 4 °C with anti-GST (M20007, Abmart, Shanghai, China), anti-K14 (ab119695, Abcam, Cambridge, MA, USA), or anti-K17 (4543, Cell Signaling Technology, Danvers, MA, USA) primary antibodies. After incubation with HRP-conjugated secondary antibodies (Proteintech, Wuhan, China) at room temperature for 1 h, signals were detected by chemiluminescence (E423, Vazyme, Nanjing, China).

### 2.5. Establishment of a Psoriasis Mouse Model

Mice were anesthetized by intraperitoneal injection of 0.7% sodium pentobarbital (10 μL/g). Dorsal hair was removed using an electric clipper. For C57BL/6J mice, residual hair was further removed using depilatory cream. For primary immunization, a mixture of the corresponding antigen emulsified with an equal volume of complete Freund’s adjuvant (CFA) was injected intradermally into the dorsal skin. Booster immunizations were administered 3–4 weeks later using the same antigen emulsified with incomplete Freund’s adjuvant (IFA) via subcutaneous injection. For the adjuvant control group, mice were injected with CFA via the intradermal route or with IFA via the subcutaneous route. Approximately 25 µL was injected per site, for a total volume of 200 µL per mouse. For control groups, BALB/c mice were only anesthetized and subjected to dorsal hair clipping, while C57BL/6J control mice additionally received depilatory cream treatment. For the IMQ control group, BALB/c mice received daily topical application of 10 mg IMQ to the tail base, whereas C57BL/6J mice received 62.5 mg IMQ daily to the dorsal skin for 7 consecutive days. For each mouse strain, five independent experiments were performed (two with GST-tagged proteins and three with TF-tagged proteins). In each experiment, the control and tag control groups each comprised 5 mice, while the K14 and K17 immunization groups each comprised 10 mice.

### 2.6. Hematoxylin and Eosin Staining

Tissue samples were collected from lesional skin of symptomatic mice (tail from BALB/c, dorsal skin from C57BL/6J), fixed overnight in 4% paraformaldehyde, dehydrated, and embedded in paraffin. For comparison, tissue samples from control mice were collected from the corresponding anatomical sites. Sections were sliced at a thickness of 5–7 μm and subjected to hematoxylin and eosin (H&E) staining according to the manufacturer’s instructions (Servicebio, Wuhan, China).

### 2.7. Antibody Detection

Blood (50 µL) was collected from the tail tip and centrifuged at 1500× *g* for 10 min after storage at 4 °C for 2–4 h to obtain serum. ELISA was performed using the Antibody Pair Auxiliary Kit (PK10029, Proteintech). Briefly, GST-K14 and GST-K17 (4 µg/mL in coating buffer) were immobilized overnight at 4 °C, followed by blocking with ELISA Universal Diluent for 1 h at room temperature. Serially diluted serum samples were incubated for 2 h at room temperature. After washing, HRP-conjugated Goat Anti-Mouse IgG(H + L), HRP-conjugated Goat Anti-Mouse IgM (Proteintech, SA00012-6), Goat Anti-Mouse IgG1 (Abcam, ab97240), or Goat Anti-Mouse IgG2b (M32407, Invitrogen, Waltham, MA, USA) secondary antibodies were added at 100 μL per well and incubated at room temperature for 1 h. TMB substrate was added for approximately 10–15 min in the dark, and the reaction was stopped with stop buffer. OD was read at 450 nm. Antibody titers were determined using the positive/negative (P/N) ratio method.

### 2.8. Cytokine Analysis by Cytometric Bead Array (CBA)

Blood (100 μL) was collected from the orbital venous plexus of each mouse into a 1.5 mL centrifuge tube containing heparin (IR9174, Solarbio, Beijing, China), immediately mixed by inversion, allowed to stand at 4 °C for 4 h, and then centrifuged at 500× *g* for 5 min to transfer the plasma to a new centrifuge tube. Cytokine concentrations (IL-10, IL-4, IFN-γ, IL-6, TNF, and IL-17A) were measured using the Mouse Th1/Th2/Th17 CBA Kit (560485, BD Biosciences, San Jose, CA, USA) according to the manufacturer’s instructions. Briefly, samples or standards were incubated with capture beads and detection reagents for 2 h at room temperature in the dark. After washing, beads were acquired on a flow cytometer and analyzed using BD CBA software (FCAP Array v3). Standard curves were generated from serial dilutions of the provided standards, and only curves with R^2^ ≥ 0.99 were accepted for quantification.

### 2.9. T Cell Functional Assay

Cell culture plates were coated with 2 µg/mL anti-CD3 antibody (14003286, Invitrogen) overnight at 4 °C for the positive control. Lymph node and spleen cells were seeded at 1 × 10^6^ cells/well in 1640 medium supplemented with 10% heat-inactivated FBS, 50 U/mL IL-2, 1% Pen-Strep, and 50 µM β-ME. Cells were stimulated with 1 μg/mL K14/K17 protein and 5 µg/mL anti-CD28 antibody (14028186, Invitrogen) at 37 °C in 5% CO_2_ for 48 h. Culture supernatants were then collected, and IFN-γ levels were measured using an IFN-γ ELISA antibody pair (PK00132, Proteintech) according to the manufacturer’s protocol. IFN-γ levels were calculated and graphed using GraphPad Prism.

### 2.10. BCA Protein Assay

Protein concentrations were determined using a BCA protein assay kit (A55865, Thermo Fisher Scientific, Waltham, MA, USA) following the manufacturer’s protocol. Briefly, samples and bovine serum albumin (BSA) standards (0–2000 µg/mL) were incubated with the BCA working reagent at 37 °C for 30 min, and the absorbance was measured at 562 nm. Only standard curves with an R^2^ value ≥0.99 were accepted for quantification.

### 2.11. Statistical Analysis

All data are presented as mean ± standard error of the mean (SEM). Statistical analysis was performed using GraphPad Prism 8.0. Comparisons between the control and treatment groups were conducted using one-way analysis of variance (ANOVA) followed by Tukey’s post hoc test or unpaired *t*-test, as appropriate.

## 3. Results

### 3.1. Expression and Purification of K14/K17 Proteins

**Figure 1:**
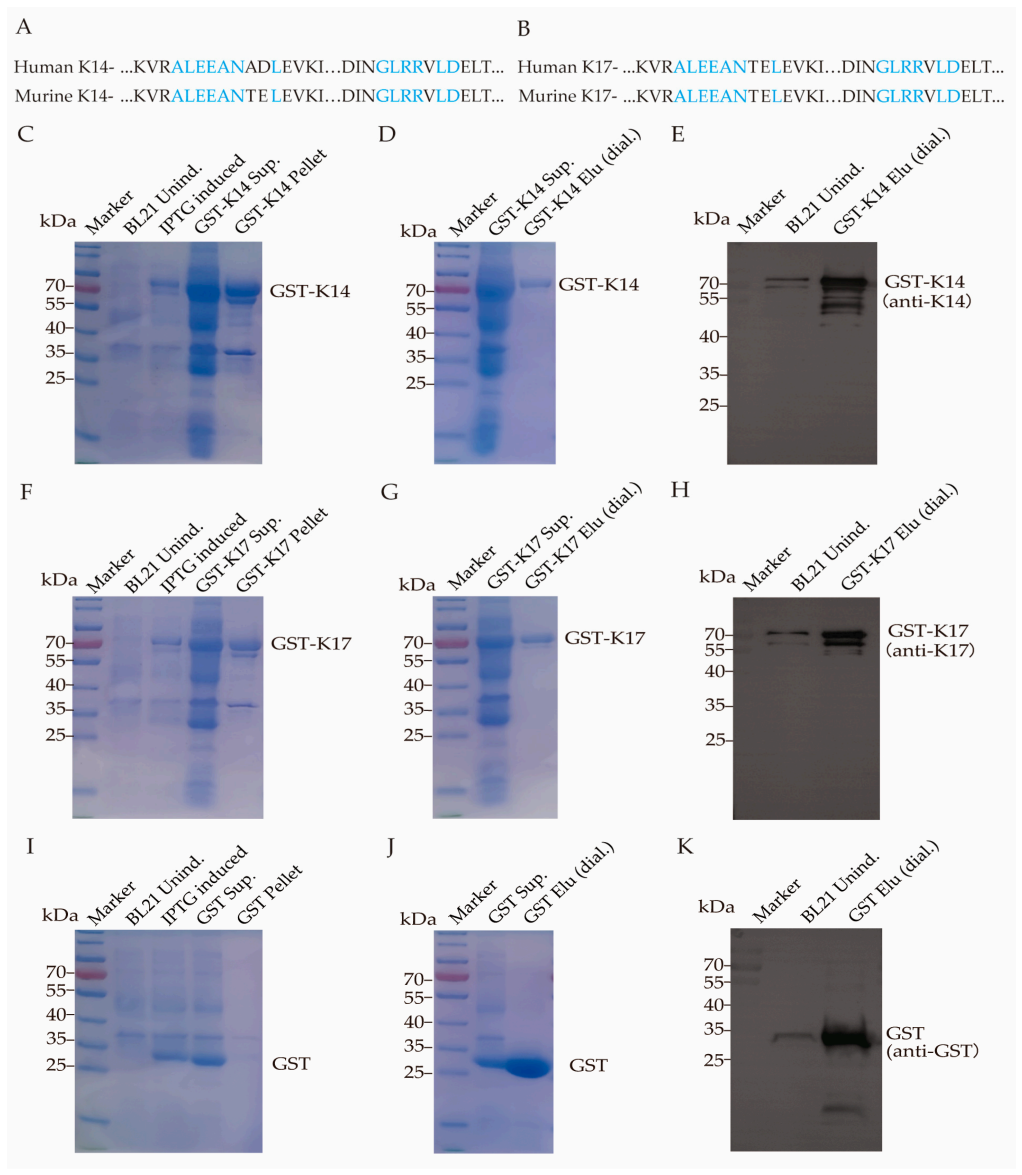
Expression and purification of Glutathione S-transferase (GST)-tagged keratin (K)14 and K17 proteins. Sequence analysis of shared “ALEEANxxL” and “GLRRxLD” motifs in human and murine K14 (**A**) and K17 (**B**) proteins with M protein was performed using DNAMAN. (**C**) SDS-PAGE analysis of GST-K14 protein expression. (**D**) SDS-PAGE analysis of purified GST-K14 protein. (**E**) Validation using a K14 protein-specific antibody. (**F**) SDS-PAGE analysis of GST-K17 protein expression. (**G**) SDS-PAGE analysis of purified GST-K17 protein. (**H**) Validation using a K17 protein-specific antibody. (**I**) SDS-PAGE analysis of GST protein expression. (**J**) SDS-PAGE analysis of purified GST protein. (**K**) Validation using a GST protein-specific antibody. Representative immunoblot results are shown. Coomassie Brilliant Blue staining: 10–50 µg protein/lane. Western blot: 0.5–1 µg protein/lane. All experiments were repeated at least three times. Abbreviation: Unind, uninduced; Sup, supernatant; Elu (dial.), Elution (dialyzed).

It is accepted that aberrantly expressed K17 serves as one of the potential autoantigens associated with psoriasis [7,8]. Its homologous K14 shares common characteristic sequences and is also aberrantly expressed in psoriasis [10]; we chose both K14 and K17 as immunogens to investigate whether active immunization against these keratins induces psoriasis-like skin inflammation. Through sequence alignment, we found that both human and murine K14 and K17 contain the characteristic motifs “ALEEANxxL” and “GLRRxLD” identical to those found in the virulence factor M protein of group A β-hemolytic *Streptococcus* (Figure 1A,B), indicating that they may trigger autoimmune responses through molecular mimicry. To establish a mouse model for antigen-specific psoriasis, we set out to immunize mice with K14/K17. First, we expressed K14/K17 proteins using a prokaryotic expression system, purified them by affinity chromatography, and performed Western blot validation. As shown in Figure 1C–K, we successfully obtained GST-K14 and GST-K17 fusions, and GST as a control.

During the purification of K14 and K17 proteins, we found that tag removal resulted in substantial protein loss. To exclude the potential influence of the tags, in addition to purifying GST-tagged proteins, we also purified TF-tagged proteins for mouse immunization, with the corresponding tags used as controls. As shown in Supplementary Figure S1A,B, we expressed and purified TF-K14 and TF-K17 fusion proteins, as well as the TF protein as a control (Supplementary Figure S1C). TF-K14 and TF-K17 were validated by Western Blot using specific antibodies (Supplementary Figure S1D,E).

### 3.2. K14/K17-Immunized Mice Exhibited Psoriasis-like Symptoms

**Figure 2:**
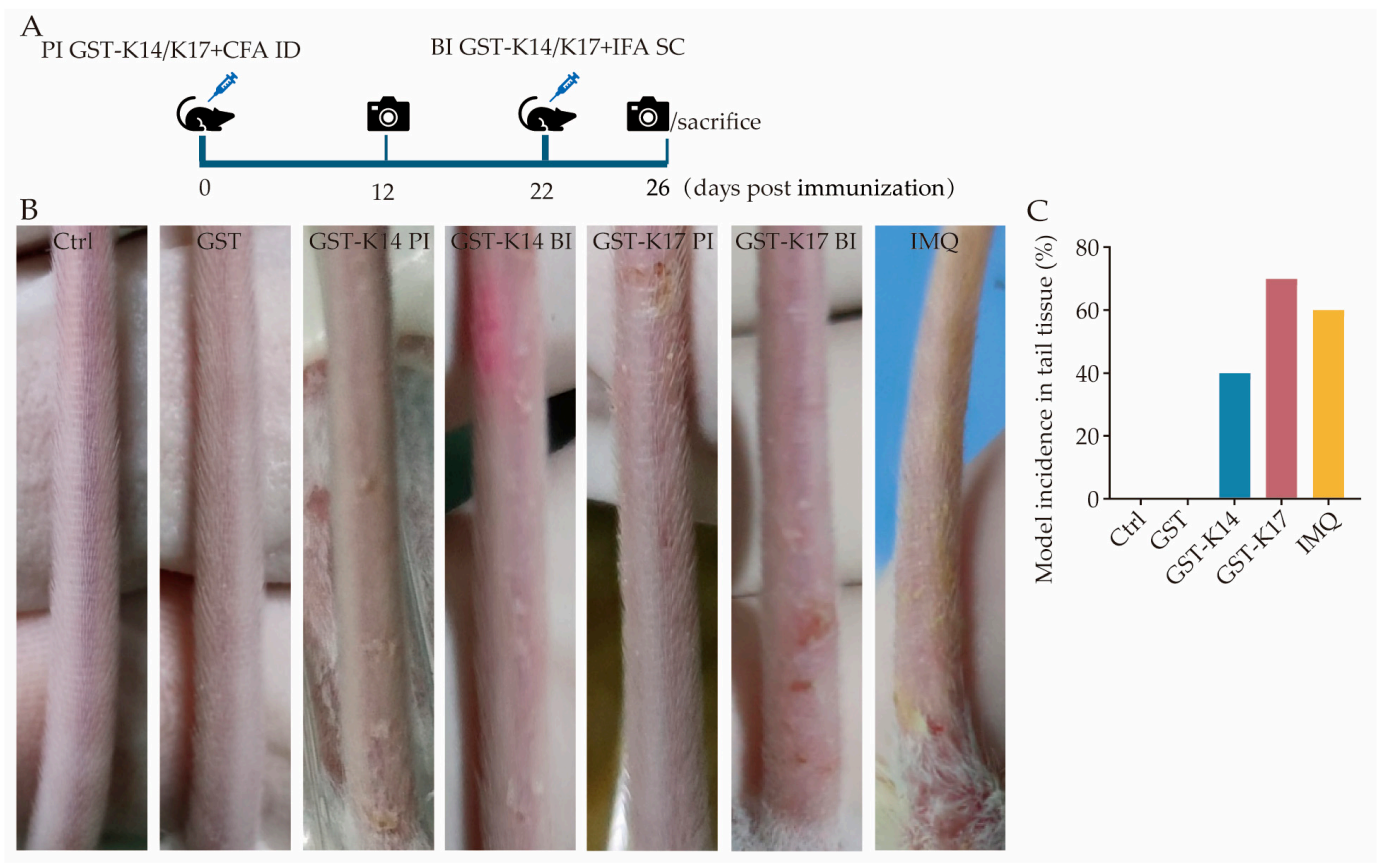
Psoriasis-like manifestations in BALB/c mice immunized with GST-K14/K17. (**A**) Schematic diagram of the immunization strategy with GST-K14/K17 proteins. (**B**) Symptoms observed in mice following immunization with GST-K14/K17 proteins. For primary immunization, 6-week-old mice were intradermally injected with 350 μg of GST (*n* = 5), GST-K14 (*n* = 10), or GST-K17 (*n* = 10). For booster immunization, mice were subcutaneously injected with 450 μg of GST, GST-K14, or GST-K17. Photographs of mouse tails were taken at the time points indicated in panel A. Representative results are shown, and the model establishment experiment was independently repeated at least twice with similar results. (**C**) Quantification of symptomatic mice incidence from (**B**) following primary immunization with GST-K14/K17 proteins. Abbreviation: Pl, primary immunization; BI, booster immunization; ID, intradermal injection; SC, subcutaneous injection; CFA, complete Freund’s adjuvant; IFA, incomplete Freund’s adjuvant.

We injected CFA-emulsified GST, GST-K14, or GST-K17 proteins into the back of female BALB/c mice via the intradermal route, or applied IMQ to the tail base as a control, and examined the mice for skin lesions of psoriasis every day post-immunization (Figure 2A). As a result, the onset of extra-injection-site symptoms was first observed on day 8 post primary immunization. Subsequently, both the incidence and the severity of the symptoms increased daily. By day 12, at least 4 out of 10 mice had developed psoriatic lesions, displaying punctate or patchy yellow-to-white crusts and scales on the tail (Figure 2B). These lesions persisted up to the time of the second immunization. The proportion of mice with psoriasis symptoms reached 40% (4/10) in the GST-K14 group and 70% (7/10) in the GST-K17 group (Figure 2C) after primary immunization (PI). Three weeks post primary immunization, we administered a booster immunization (BI) into the backs of the mice via subcutaneous injection. Four days post-booster, the psoriasis lesions on the tail of the diseased mice exhibited exacerbated erythema and scaling (GST-K14 BI and GST-K17 BI in Figure 2B). These results indicate that injection of mice with purified K14 and K17 can induce psoriasis-like symptoms in BALB/c mice.

To further determine the role of K14 and K17 in inducing psoriasis, we performed parallel experiments, immunizing mice with TF-K14 and TF-K17 fusion proteins (Supplementary Figure S2A). Compared with the controls, TF-K14-immunized mice developed multiple visible plaques and scales on the tail (non-injection site) by day 8 post immunization, while TF-K17-immunized mice exhibited psoriasis-like symptoms by day 11 post immunization (Supplementary Figure S2B). These symptoms persisted until day 35 post-immunization, lasting for more than 27 days, which was significantly longer than that of the IMQ-induced model with short-term clinical signs. Our data show that the proportion of mice developing symptoms in both the TF-K14 and TF-K17 groups was 40% (Supplementary Figure S2C). These results indicate that regardless of GST or TF tags, both K14 and K17 proteins induced psoriasis-like symptoms in BALB/c mice, suggesting that these two keratins themselves possess the ability to drive aberrant immune responses and elicit psoriasis phenotypes.

### 3.3. Pathological Manifestations in BALB/c Mice Immunized with K14/K17

**Figure 3:**
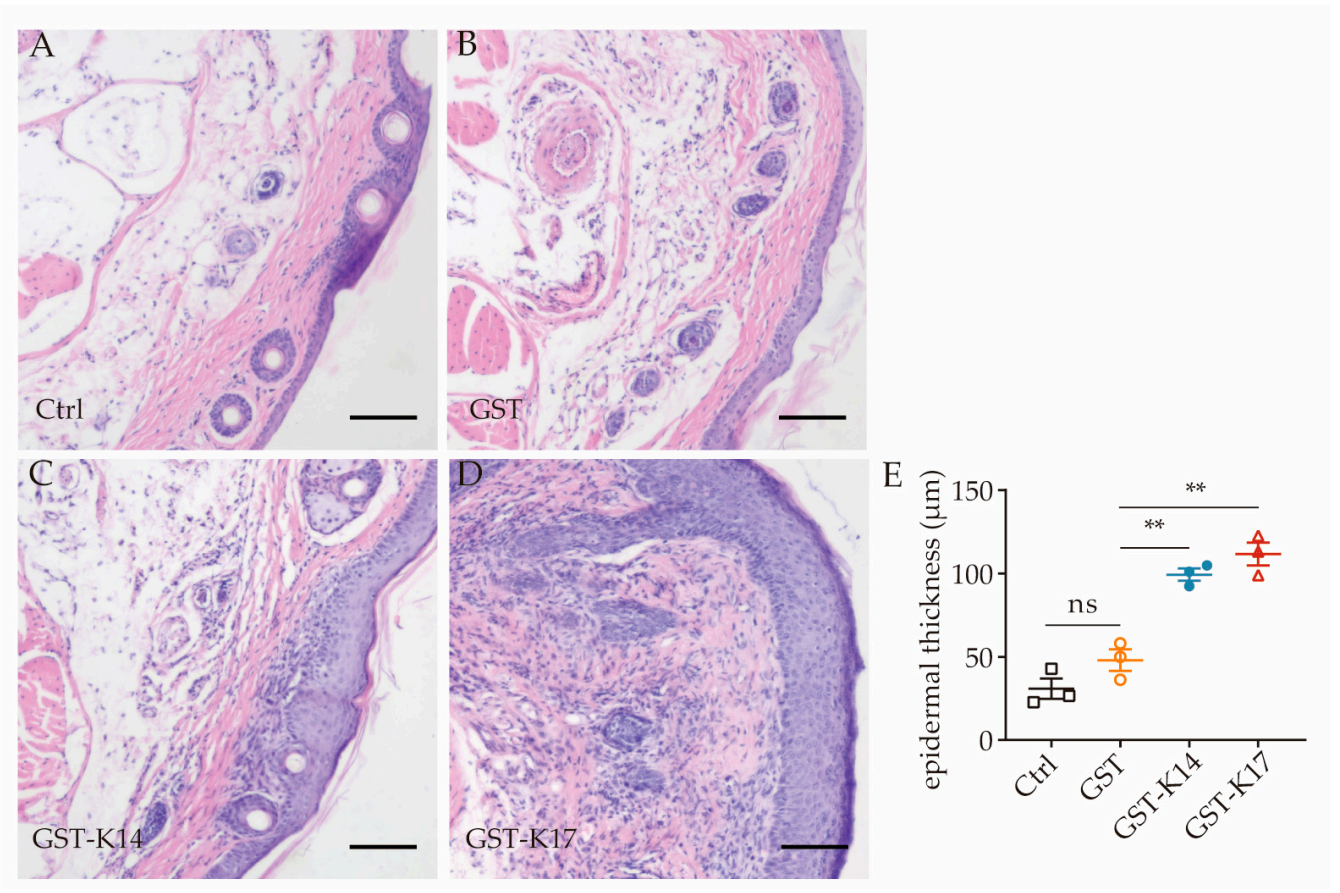
Psoriasis-like pathological manifestations in the tail of BALB/c mice immunized with GST-K14/K17. (**A**–**D**) H&E staining of symptomatic tails from BALB/c mice. Representative staining results are shown; scale bar = 100 μm. (**E**) Quantitative analysis of tail epidermal thickness based on morphometric measurements from three different visual microscopic fields. Data were from three independent experiments and presented as mean ± SEM, analyzed by one-way ANOVA, ** *p* < 0.01, ns (not significant), *n* ≥ 3.

Histopathological analysis of the tail lesions of BALB/c mice with psoriasis-like clinical signs reveals that the crusted and scaly areas on the tails of immunized mice exhibited significant epidermal hyperplasia and inflammatory cell infiltration (Figure 3A–D), resembling the pathological features of psoriasis. Measurement of epidermal thickness in three different visual fields under the microscope indicates that compared to the GST controls, GST-K14 and GST-K17 immunized mice displayed significantly thickened tail epidermis (Figure 3E).

**Figure 4:**
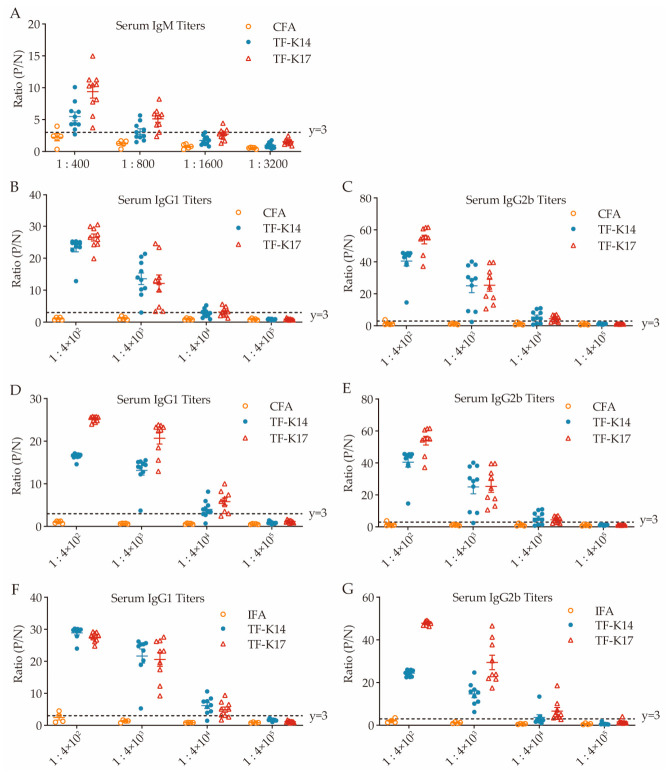
A high level of specific antibodies in the serum of BALB/c mice immunized with TF-K14/K17. (**A**) Detection of specific IgM against TF-K14/K17 in mouse serum 7 days post primary immunization using ELISA. (**B**,**C**) Detection of specific IgG1 (**B**) and IgG2b (**C**) against TF-K14/K17 in mouse serum 14 days post primary immunization using ELISA. (**D**,**E**) Detection of specific IgG1 (**D**) and IgG2b (**E**) against TF-K14/K17 in mouse serum 21 days post primary immunization using ELISA. (**F**,**G**) Detection of specific IgG1 (**F**) and IgG2b (**G**) against TF-K14/K17 in mouse serum 3 days post booster immunization using ELISA. GST-K14 and GST-K17 (4 µg/mL) were coated overnight at 4 °C. Data are presented as mean ± SEM, *n* ≥ 4.

To determine the dynamics in antibody production, we examined the antibody subtypes in mice immunized with TF-K14 and TF-K17. Our data show that the titers of IgM against TF-K14 and TF-K17 in mouse serum were 1:800 and 1:1600, respectively, at 7 days post-primary immunization (Figure 4A). The titers of both IgG1 and IgG2b subtypes against TF-K14 and TF-K17 in mouse serum exceeded 1:4 × 10^4^ at 14 days, 21 days post primary immunization, and 3 days post booster immunization (Figure 4B–G). These results indicate that immunization of mice with K14 and K17 could induce a robust humoral immune response. The high levels of both IgG1 (typically associated with a Th2 response) and IgG2b (typically associated with a Th1 response) suggest that this immunization strategy elicited balanced and potent mixed Th1/Th2 immune responses.

### 3.4. Expression of Inflammatory Cytokines in BALB/c Mice Immunized with K14/K17

**Figure 5:**
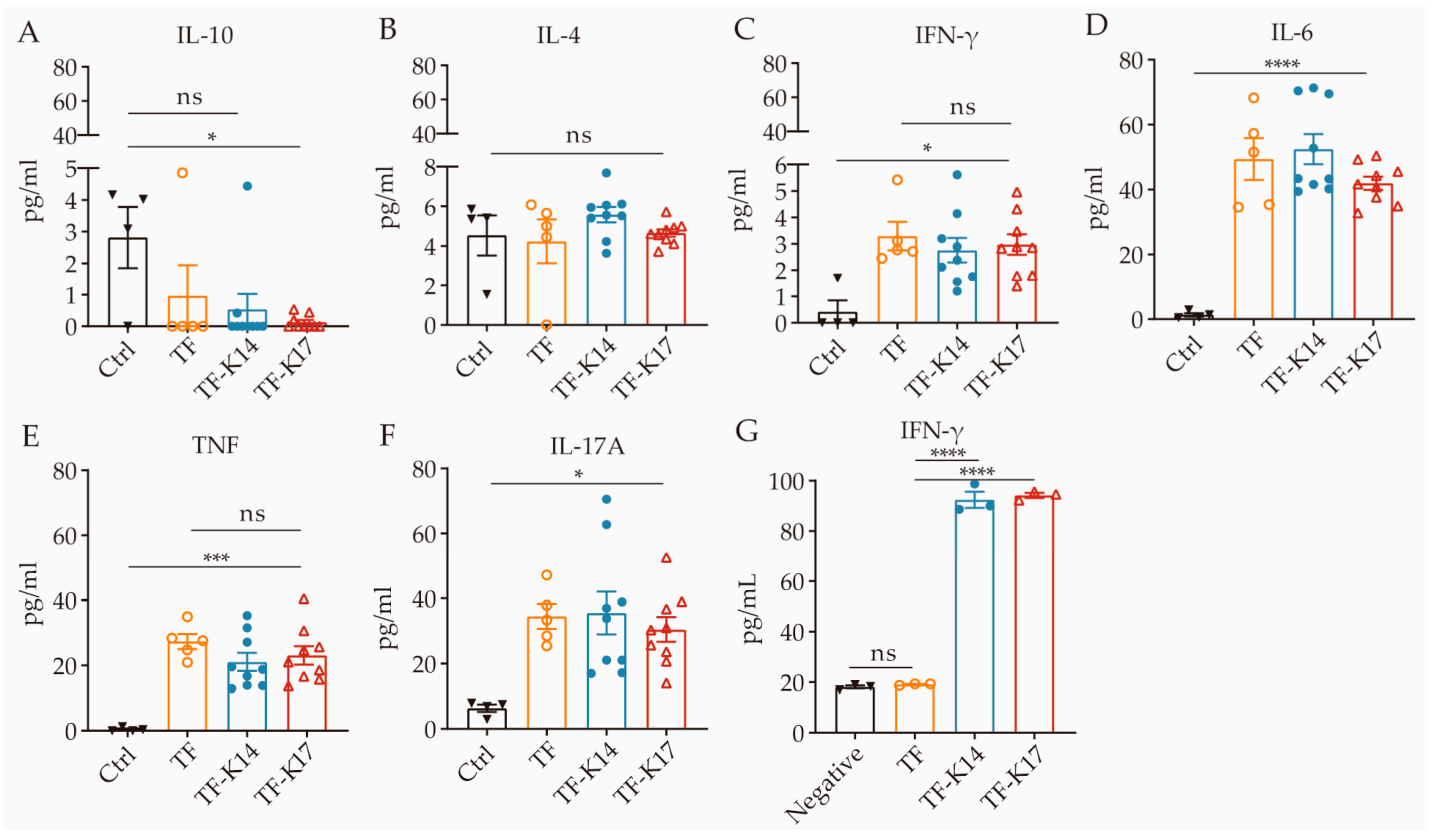
Inflammatory cytokine production in BALB/c mice following immunization with TF-K14/K17. The levels of IL-10 (**A**), IL-4 (**B**), IFN-γ (**C**), IL-6 (**D**), TNF (**E**), and IL-17A (**F**) in the blood of mice immunized with TF, TF-K14, TF-K17 and PBS as controls were detected using a CBA kit (BD Biosciences, San Jose, CA, USA) 7 days post-primary immunization. All immunized mice (including asymptomatic K14/K17 animals) were included in the analysis. *n* ≥ 4. (**G**) T cells from the inguinal lymph nodes and spleen of mice 7 days post booster immunization were cultured in the presence or absence of TF/GST-K14/GST-K17 (1 μg/mL) for 48 h, and IFN-γ in the culture supernatant was detected by ELISA. Data are presented as mean ± SEM, analyzed by one-way ANOVA, * *p* < 0.05, *** *p* < 0.001, **** *p* < 0.0001, ns (not significant).

To further evaluate the impact of K14/K17 immunization on inflammatory response in mice, we measured the levels of inflammatory cytokines in the blood of BALB/c mice 7 days post immunization. As shown in Figure 5A–C, although IL-10, IL-4 and IFN-γ levels were detected by CBA and showed statistically significant differences between groups, these values were below the standard range (20 pg/mL) and were therefore considered to lack biological or clinical significance. In contrast, immunization markedly elevated systemic IL-6, TNF, and IL-17A levels in the blood of mice at 7 days post immunization (Figure 5D–F). Notably, although these pro-inflammatory cytokines were elevated in the TF, TF-K14, and TF-K17 groups compared to the naive control group, there were no significant differences between the TF-K14 and TF-K17 groups and the TF control group. To further characterize the inflammatory profile, future studies focusing on symptomatic individuals, additional time points, or local tissue responses are required. We further investigated IFN-γ expression by antigen-specific T cells from mice boosted with TF-K14/K17. As shown in Figure 5G, the levels of IFN-γ in the cultures of lymph node and spleen cells from K14/K17-booster mice were greater than controls post stimulation with GST-K14/K17 proteins, which suggests that K14/K17 immunization induced an adaptive immune response.

### 3.5. Psoriasis-like Symptoms in C57BL/6J Mice Following Immunization with K14/K17

**Figure 6:**
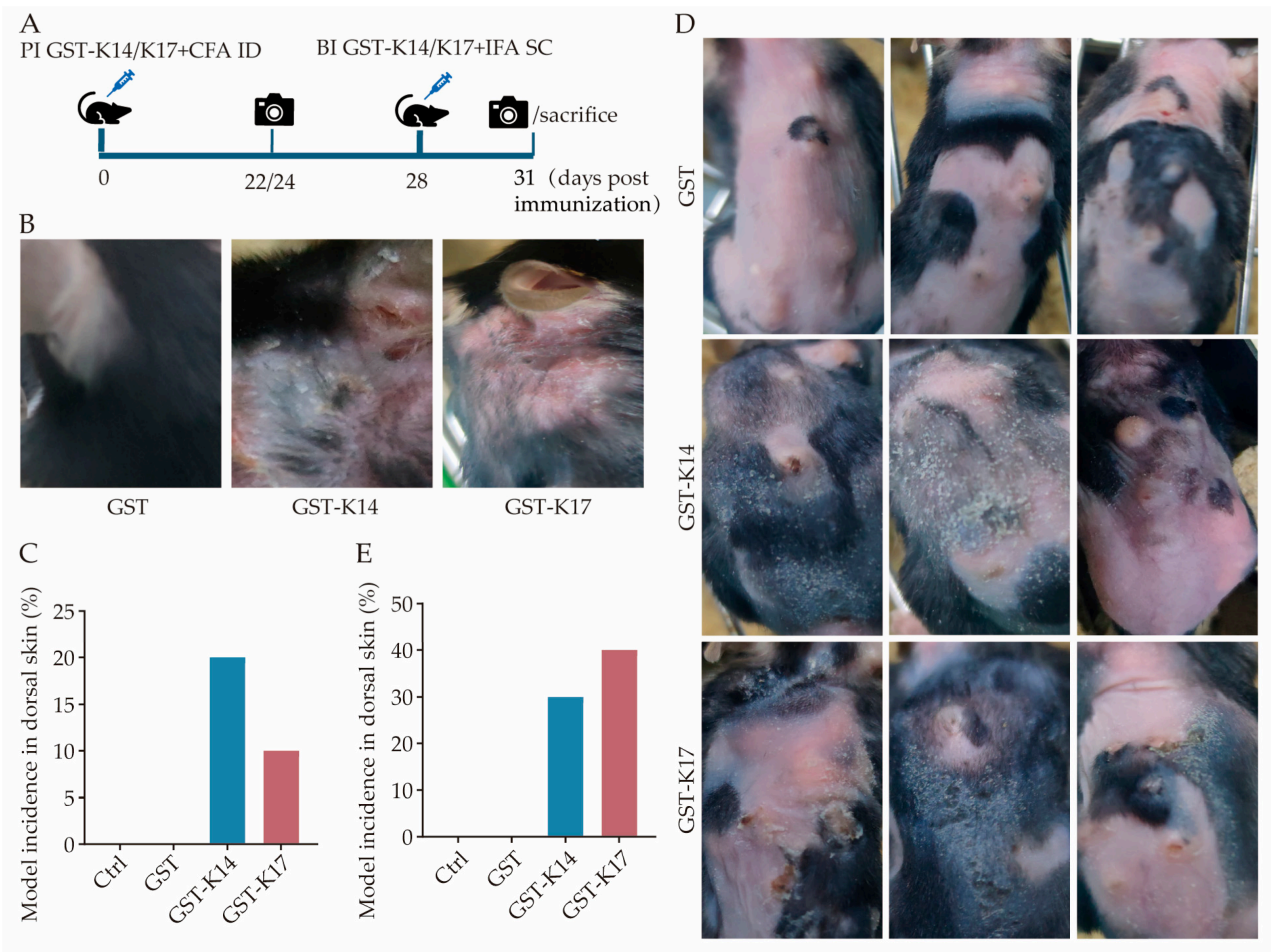
Psoriasis-like symptoms in C57BL/6J mice following immunization with GST-K14/K17. (**A**) Schematic diagram of the mouse immunization with GST-K14/K17 proteins. (**B**) Psoriasis-like symptoms in mice 22–24 days post-primary immunization with GST-K14/K17 proteins. For primary immunization, 6-week-old mice were intradermally injected with 350 μg of GST-K14 (*n* = 10), GST-K17 (*n* = 10), or GST (*n* = 5) as controls. Photographs of mouse backs were taken at the time points as described above in (**A**). (**C**) The incidence of psoriasis-like symptoms in mice from (**B**) 22–24 days post primary immunization with GST-K14/K17 proteins. (**D**) Symptoms observed in mice 3 days post booster immunization with GST-K14/K17 proteins. For booster immunization, mice were subcutaneously injected with 450 μg of GST-K14, GST-K17, and GST, respectively. Data are representative of at least two experiments with similar results. (**E**) The incidence of psoriasis-like symptoms in mice from (**D**) 3 days post booster immunization with GST-K14/K17 proteins.

To determine whether the antigen-specific psoriasis caused by K14 and K17 vaccination of BALB/c could be generalized to mice with other genetic backgrounds, we injected C57BL/6J mice with K14 and K17 fusions, and examined the skin lesions in mice post K14 and K17 injection (Figure 6A). As a result, mice injected with K14 and K17 proteins displayed erythema and scaling on the backs (non-injection site) at 22–24 days after the primary immunization (Figure 6B), which lasted for approximately 5 days. In contrast, no clinical symptoms were observed in the control group. These features are consistent with the clinical characteristics of psoriasis. The incidence rate in GST-K14-vaccinated mice was 20% (2/10), and in the GST-K17 group, 10% (1/10) (Figure 6C). Three days post booster immunization, mice immunized with GST-K14 and GST-K17 developed extensive white scales on the back (non-injection site) compared with the control groups, and the scales persisted for approximately 5 days (Figure 6D). The incidence rates of GST-K14 and GST-K17-induced psoriasis in mice increased up to 30% (3/10) and 40% (4/10), respectively (Figure 6E), indicating that C57BL/6J mice displayed psoriasis-like skin lesions post injection with K14 and K17 proteins. These results suggest that K14 and K17-induced psoriasis in mice was independent of their genetic backgrounds.

When we used the same immunization regimen as indicated in Supplementary Figure S3A, no skin symptoms in mice were observed 21 days post primary immunization. However, four days post booster immunization, the mice injected with TF-K14 and TF-K17 displayed abundant white scales on the backs (non-injection site) that lasted for approximately 5 days (Supplementary Figure S3B). These observations were consistent with the symptoms observed following GST-K14/K17 booster immunization. The incidence rates of psoriasis in both TF-K14 and TF-K17-injected mice were 50% (Supplementary Figure S3C). These results indicate that the K14 and K17 proteins can successfully induce psoriasis-like skin lesions in C57BL/6J mice, independent of their fusion tags.

### 3.6. Pathological Skin Changes in C57BL/6J Mice Immunized with K14/K17

**Figure 7:**
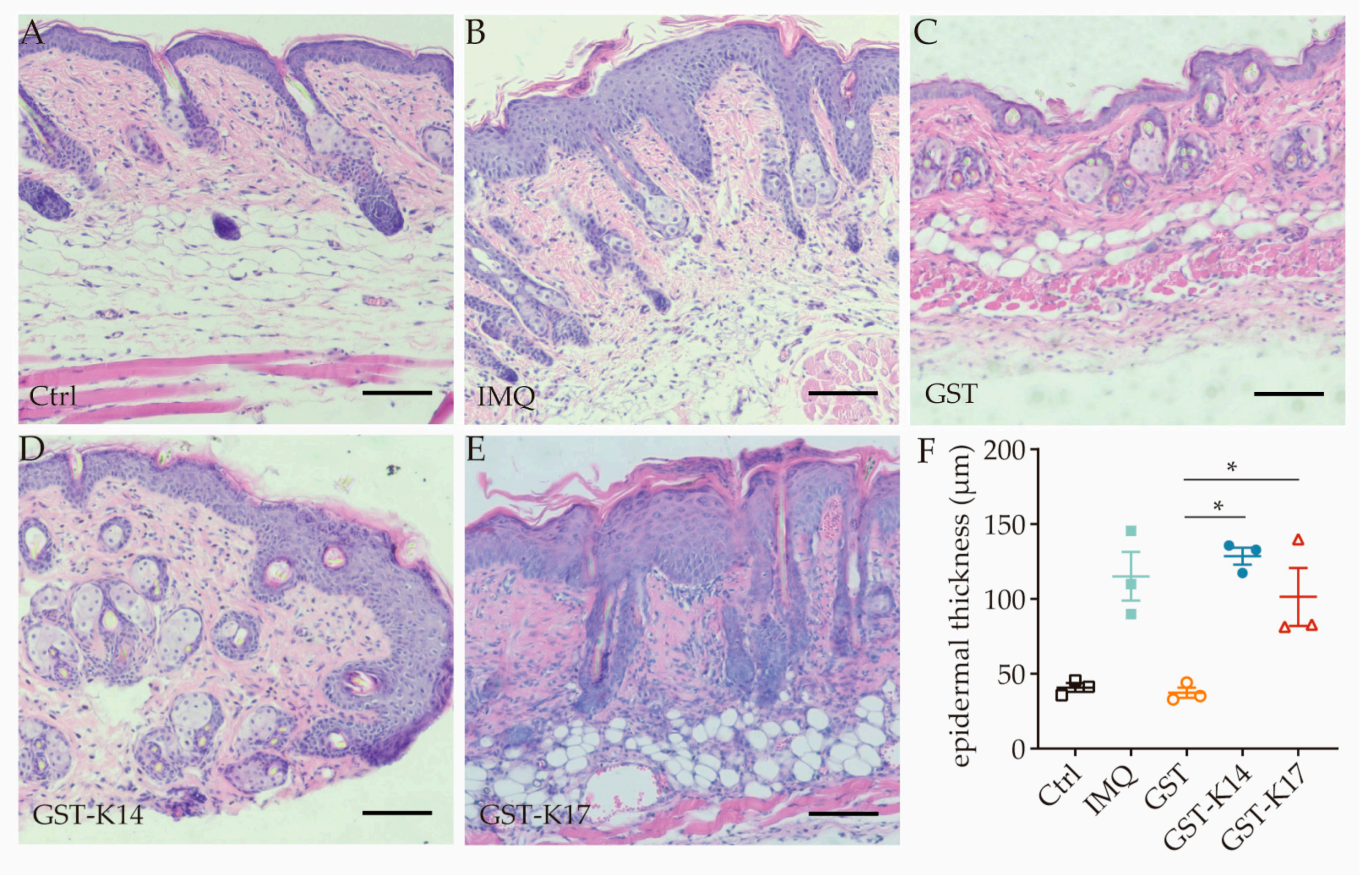
Psoriasis-like skin lesions on the back of C57BL/6J mice immunized with GST-K14 and GST-K17. (**A**–**E**) H&E staining of lesional skin on the back of C57BL/6J mice. Data are representative of three experiments with similar results. The light green color in the background of the H&E images is an optical artifact arising from the microscope imaging settings (color balance). Scale bar = 100 μm. (**F**) Comparison of epidermal thickness of skin on the back of mice. Three different visual microscopic fields for each group were measured and analyzed. Data were from three independent experiments and presented as mean ± SEM, analyzed by unpaired *t*-tests, * *p*< 0.05, *n* ≥ 3.

We performed histopathological examination of the skin on the backs of C57BL/6J mice and found that mice immunized with GST-K14/K17 exhibited white scale lesions including epidermal thickening and inflammatory cell infiltrations, similar to that of the IMQ-induced psoriasis model and consistent with the core histological changes of psoriasis (Figure 7A–F).

**Figure 8:**
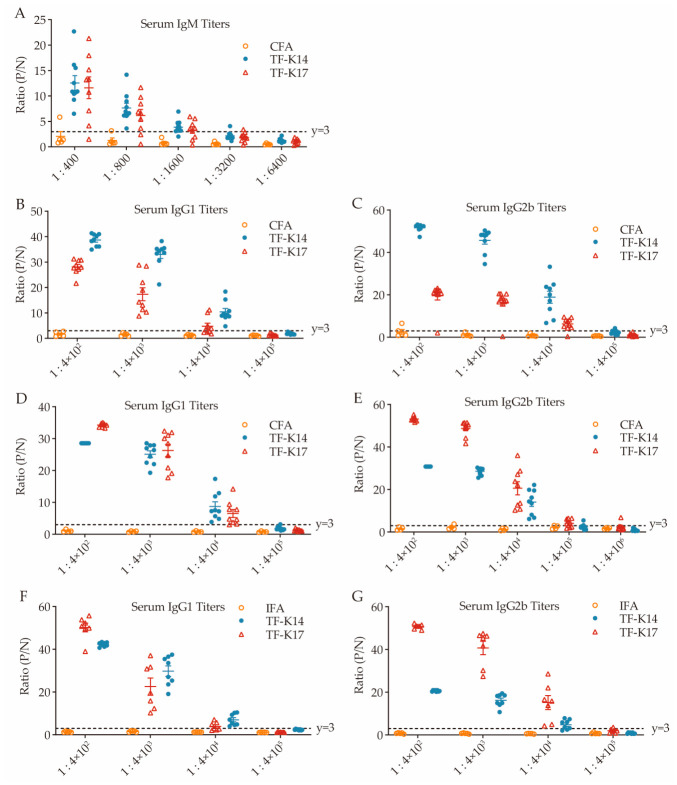
High levels of specific antibodies in the serum of C57BL/6J mice immunized with TF-K14/K17. (**A**) Detection of specific IgM against TF-K14/K17 in mouse serum 7 days post-primary immunization using ELISA. (**B**,**C**) Detection of specific IgG1 (**B**) and IgG2b (**C**) against TF-K14/K17 in mouse serum 14 days post-primary immunization using ELISA. (**D**,**E**) Detection of specific IgG1 (**D**) and IgG2b (**E**) against TF-K14/K17 in mouse serum 21 days post-primary immunization using ELISA. (**F**,**G**) Detection of specific IgG1 (**F**) and IgG2b (**G**) against TF-K14/K17 in mouse serum 3 days post booster immunization using ELISA. GST-K14 and GST-K17 (4 µg/mL) were coated overnight at 4 °C. Data are presented as mean ± SEM, *n* ≥ 4.

Furthermore, we analyzed the dynamic changes in anti-K14/K17 antibodies in immunized C57BL/6J mice. Our data show that the titers of IgM against TF-K14/K17 in mouse serum reached 1:3200 at 7 days post-primary immunization (Figure 8A). The levels of both IgG1 and IgG2b against TF-K14/K17 in mouse serum exceeded 1:4 × 10^4^ at 14 days, 21 days post primary immunization, and 3 days post booster immunization (Figure 8B–G). These results indicate that K14 and K17 were able to simultaneously induce high levels of both IgG1 and IgG2b against K14/K17, suggesting that K14/K17 elicit a balanced and robust Th1/Th2 immune response.

### 3.7. Levels of Inflammatory Cytokines in C57BL/6J Mice Post Immunization with K14/K17

**Figure 9:**
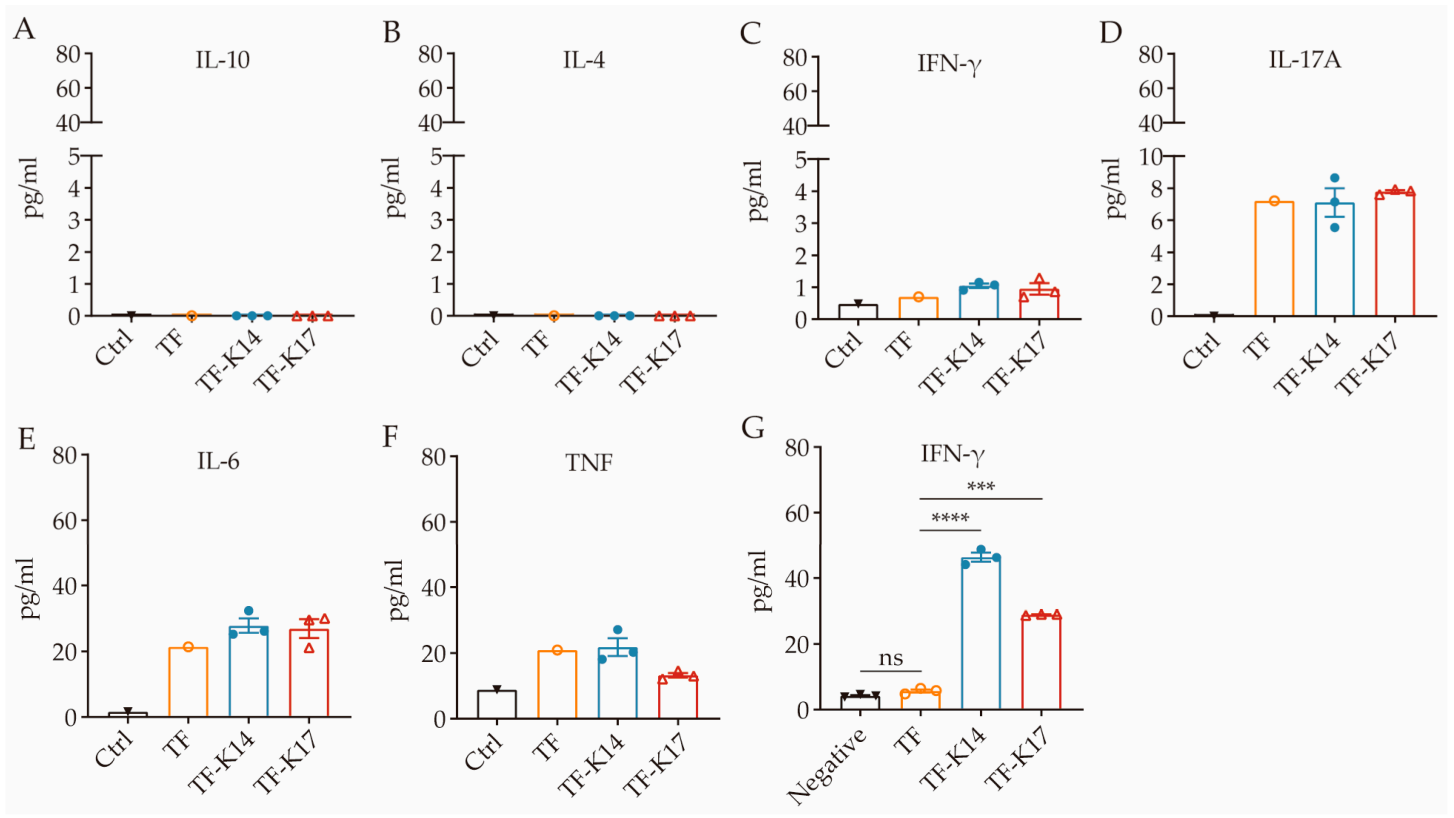
Inflammatory cytokines in C57BL/6J mice following immunization with TF-K14/K17. Plasma levels of IL-10 (**A**), IL-4 (**B**), IFN-γ (**C**), IL-17A (**D**), IL-6 (**E**) and TNF (**F**) were detected using a CBA kit in mice (Ctrl, 3 mice; TF, 5 mice; TF-K14, 15 mice and TF-K17, 15 mice) 7 days post-primary immunization. Each symbol represents a pooled sample from 3 (Control) or 5 (TF/TF-K14/TF-K17) mice; n indicates the total number of mice per group. All immunized mice (including asymptomatic K14/K17 animals) were included in the analysis. (**G**) T cells isolated from the inguinal lymph nodes and spleen of mice 10 days post booster immunization were cultured in the presence or absence of TF/GST-K14/GST-K17 (1 μg/mL) for 48 h, and IFN-γ levels in the culture supernatant were measured by ELISA. Data are presented as mean ± SEM, analyzed by one-way ANOVA, *** *p* < 0.001, **** *p* < 0.0001, ns (not significant).

To evaluate the inflammatory response in C57BL/6J mice to K14/K17 immunization, we measured inflammatory cytokines in the blood of mice 7 days post immunization. As a result, IL-10, IL-4, IFN-γ and IL-17A in the peripheral blood from mice were undetectable or below the standard range and considered insignificant (Figure 9A–D). In contrast, the levels of IL-6 and TNF in mice increased post-immunization (Figure 9E,F). Notably, although these pro-inflammatory cytokines were elevated in the TF, TF-K14, and TF-K17 groups compared to the naive control group, there were no significant differences between the TF-K14 and TF-K17 groups and the TF control group. We further examined antigen-specific T-cell responses in mice immunized with TF-K14 or TF-K17. Lymphocytes and splenocytes from immunized mice secreted significantly higher levels of IFN-γ upon antigenic stimulation compared with those from control mice (Figure 9G), indicating the generation of K14/K17-specific T cell responses.

## 4. Discussion

Psoriasis is an immune-mediated inflammatory skin disease that is chronic and recurrent, affecting individuals across different regions, ethnicities, and age groups, accounting for approximately 2–3% of the global population [1,2]. Animal models for psoriasis are urgently needed to investigate the pathogenic mechanisms and treatment of psoriasis, particularly antigen-induced psoriasis animal models. However, the current lack of specific antigen-induced psoriasis animal models hinders research progress on the pathogenesis and treatment of psoriasis. In this study, we report for the first time the successful induction of psoriatic symptoms in mice through antigen-specific immunization of mice with K14 and K17, establishing a specific antigen-induced psoriasis animal model. Following immunization of mice with K14/K17 fusions, 10–70% of the mice exhibited typical psoriasis-like phenotypes, including erythema, scaling, epidermal thickening, and inflammatory cell infiltration. Additionally, antigen-specific antibody titers were elevated, along with enhanced antigen-specific T-cell responses. These results are consistent with the immunopathological characteristics of human psoriasis [14], demonstrating that K14 and K17 can induce psoriasis in mice and providing an effective animal model for in-depth research on the pathogenesis and targeted therapy of psoriasis.

Currently, IMQ-induced skin inflammation is commonly used as a psoriasis model, but it represents skin lesions caused by innate response and fails to reflect the chronic autoimmune process of psoriasis, an autoimmune disease caused by specific autoantigens [15,16]. IL-23 injection can rapidly induce skin lesions but does not recapitulate the antigen-specific mechanisms underlying disease initiation [17]. While xenotransplantation models closely resemble human psoriatic pathology, they are difficult to apply widely due to challenges in tissue acquisition, high costs, and stringent technical requirements [18,19]. Genetically engineered or spontaneous mutation models are limited to the role of single genes, failing to reflect the complexity of polygenic regulation in psoriasis, and are characterized by long construction periods and potentially atypical phenotypes [16,20,21], hindering their applications.

Furthermore, in recent years, other novel mouse models have been developed in the field of psoriasis research, primarily aimed at enhancing the relevance of models to human disease or the precision of mechanistic studies. These models mainly include: 1. Humanized immune system models [22,23] exhibit symptoms highly similar to human lesions and can recapitulate Th1/Th17 skewing, but are associated with extremely high costs and significant individual variability. 2. Antigen-specific T cell receptor (TCR) transgenic models [24] can trigger precise T cell-mediated skin inflammation under specific stimulation, but are complex to construct and often rely on exogenous challenge. 3. VEGF transgenic animal models [25] induce dermatitis primarily characterized by local vascular proliferation and inflammatory cell recruitment, failing to reflect the chronic autoimmune process of psoriasis. 4. Local gene editing models, which are relatively flexible in operation and suitable for functional validation, but typically simulate only certain aspects of the disease process [26,27].

In contrast, in this study, K14 and K17, used as exogenous autoantigens and emulsified with CFA for active immunization of mice, successfully induced typical skin lesions. Furthermore, we found that both IgG1 and IgG2b antibodies were significantly elevated in the serum of immunized mice, suggesting that a mixed Th1/Th2-type response had been elicited. Supplementary Table S1 presents a comparison between the psoriasis model established in this study and existing psoriasis disease models.

Notably, existing studies have identified differences in the roles of K14 and K17 in psoriasis. In transgenic psoriasis models, K14 serves as a promoter tool to drive gene overexpression in the basal layer of the epidermis [28,29]. Its ability to induce autoimmunity is considered a secondary injury resulting from inflammation. In contrast, K17 acts as a key effector molecule during disease progression, exerting an inflammation-amplifying effect [30,31,32], and is generally regarded as a therapeutic target rather than an initiating antigen [8]. Both approaches bypass the initial step in psoriasis pathogenesis, namely the primary recognition of autoantigens by the immune system. The present study focuses on the potential role of K14 and K17 in being recognized by T cells as autoantigens in psoriasis through molecular mimicry. This model employs K14/K17 as immunogens to provoke antigen-specific immune responses, rather than to replicate the natural events that initiate human psoriasis. The results indicate that once an immune response directed against K14/K17 is established, it is capable of inducing psoriasis-like lesions in mice. We acknowledge that the current study does not provide direct evidence for in situ recognition of endogenous K14/K17 by T cells, nor does it include T-cell depletion or blocking studies to establish causality. Future studies employing pMHC tetramer staining, T-cell transfer experiments, and blockade of immune subsets will be essential to definitively determine whether K14/K17-specific T cells are the primary drivers of pathology in this model.

IFN-γ, a key effector cytokine secreted following T cell activation, is widely recognized as a classic and reliable indicator for demonstrating antigen-specific T cell responses [33]. Therefore, we chose to measure IFN-γ levels to preliminarily verify whether successful antigen-specific T cell responses were induced following K14/K17 immunization. The results demonstrate that this model successfully addresses the fundamental defect of “lack of antigen specificity” inherent in traditional drug-induced models, such as the IMQ-induced psoriasis model. Furthermore, this model exhibited a prolonged symptom duration in BALB/c mice (up to 27 days or more), outperforming some acute models in terms of displaying the chronic course of psoriasis [11].

We observed a phenomenon worthy of further investigation: in BALB/c mice, psoriasis-like symptoms were mainly confined to the tail; in contrast, in C57BL/6J mice, the symptoms were predominantly localized to the back. These differences may not represent model deficiencies but rather reveal the interplay between genetic background and the local immune microenvironment in the pathogenesis of psoriasis. These findings appear to be consistent with the clinical phenomenon of psoriasis in humans, where lesional characteristics vary across different anatomical sites within the same patient [34]. This may suggest that the ultimate localization of K14/K17-induced lesions in this systemic autoimmune model could be profoundly modulated by local tissue properties, an observation that warrants further investigation.

This study has established a valuable psoriasis animal model that could be applied to the investigation into the mechanism of psoriasis; the model displayed variability of symptom incidence (10–70%) and duration across different mouse strains as mentioned above. This discrepancy may be attributable to differences in mouse strain and the number of immunizations. Across several independent replicate experiments, we observed that the incidence of disease in BALB/c mice was consistently above 40%. In addition, symptom duration in these mice lasted for up to 27 days. In contrast, C57BL/6J mice receiving only primary immunization exhibited relatively low and variable incidence rates (10–20%), which contributed to the overall level of instability and low reproducibility within the model. However, when C57BL/6J mice received a booster immunization, the incidence became more stable, ranging from 30% to 50%. These findings suggest that the reproducibility of the model can be substantially improved by selecting appropriate mouse strains and optimizing the immunization regimen, allowing selection of different incidences of the disease based on different mouse strains and immunization protocols.

Additionally, C57BL/6J mice exhibited a shorter duration of symptoms (lasting for approximately 5 days), suggesting a stronger self-limiting tendency, which may be related to the genetic backgrounds. Furthermore, the immunogenicity of the recombinant protein tags (GST/TF) may affect the immune response; we performed sequence homology analysis (Supplementary Figure S4A–C). The results showed that the sequence identities of GST and TF with K14 were only 3.93% and 4.5%, respectively, whereas the homology between K14 and K17 was 62.19%. The low sequence homology between the tag proteins and K14, together with the similar results obtained using different fusion tag systems (GST and TF), suggests that immunization with K14/K17 proteins is primarily responsible for the induction of psoriasis-like phenotypes in mice.

Furthermore, we acknowledge several limitations in this study. This study is primarily proof-of-concept in nature and does not provide definitive validation of the pathogenic mechanisms underlying the observed phenotype. Systematic longitudinal clinical scoring was not performed, limiting our ability to assess disease kinetics quantitatively. Additionally, we acknowledge that the applicability of the findings to male mice remains unknown and that potential sex-dependent differences in disease susceptibility or immune responses were not evaluated.

## 5. Conclusions

In this study, we have successfully established an antigen-specific psoriasis mouse model using K14/K17 proteins. This model not only recapitulates the typical clinical symptoms and pathological changes of psoriasis but also reproduces its autoimmune-related clinical features, providing a valuable experimental platform for in-depth investigation into the autoimmune mechanisms of psoriasis and for screening targeted therapeutic drugs. With further optimization and validation in the future, this model is expected to become an important tool in psoriasis research.

## Data Availability

All data generated or analyzed during this study are included in this published article. The datasets are available from the corresponding author upon reasonable request.

## Supplementary Materials

The following supporting information can be downloaded at: https://www.mdpi.com/article/10.3390/biology15151277/s1, Figure S1: Expression and Purification of TF-tagged K14 and TF-K17 Proteins; Figure S2: Psoriasis-like Symptoms in BALB/c Mice Immunized with TF-K14/K17; Figure S3: Psoriasis-like Symptoms in C57BL/6J Mice Following Immunization with TF-K14/K17; Figure S4: Sequence Homology Analysis of GST, TF, K14, and K17; Table S1: Characteristics of Major Psoriasis Mouse Models; File S1: Original Images.

## Abbreviations

The following abbreviations are used in this manuscript:

K17	Keratin 17
K14	Keratin 14
IMQ	imiquimod
GST	Glutathione S-transferase
TF	Trigger factor
CFA	Complete Freund’s adjuvant
IFA	Incomplete Freund’s adjuvant
H&E	Hematoxylin and eosin
CBA	Cytokine analysis by cytometric bead array
P/N	positive/negative
Unind.	Uninduced
Sup.	Supernatant
Elu (dial.)	Elution (dialyzed)
PI	Primary immunization
BI	Booster immunization
ID	Intradermal injection
SC	Subcutaneous injection
