# Supplementary material for: Establishment of a Keratin 14/17-Induced Antigen-Specific Psoriasis-like Mouse Model
Source: Biology (Basel). 2026 Aug 3;15(15):1277. doi: 10.3390/biology15151277 (PMC13464592; doi:10.3390/biology15151277)
Supplement: Supplementary file 1 [file biology-15-01277-s001.zip › biology-4363740-supplementary-revision.pdf]

Supplementary Materials

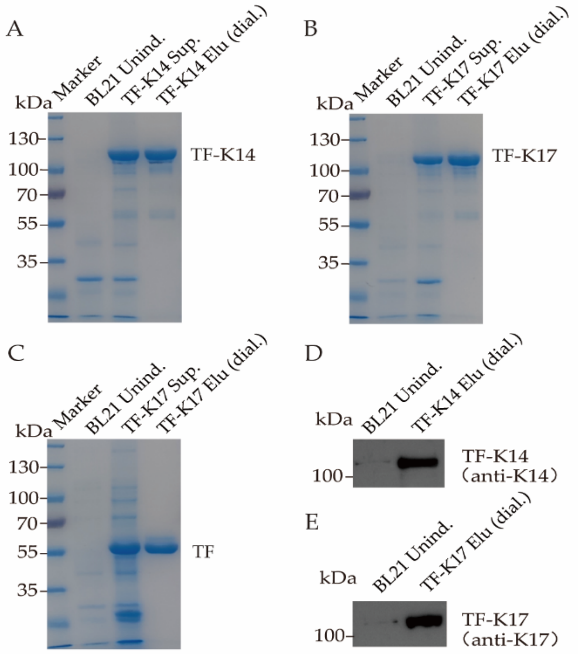

Supplementary Figure S1. Expression and purification of TF-tagged K14 and TF-K17 proteins. (A) SDS-PAGE analysis of TF-K14 protein expression and purification. (B) SDS-PAGE analysis of TF-K17 protein expression and purification. (C) SDS-PAGE analysis of TF protein expression and purification. (D&E) Validation of K14/K17 proteins using specific antibodies. Coomassie Brilliant Blue staining: 10-50 µg protein/lane. Western blot: 0.5-1 µg protein/lane. Data are representative of three experiments with similar results.

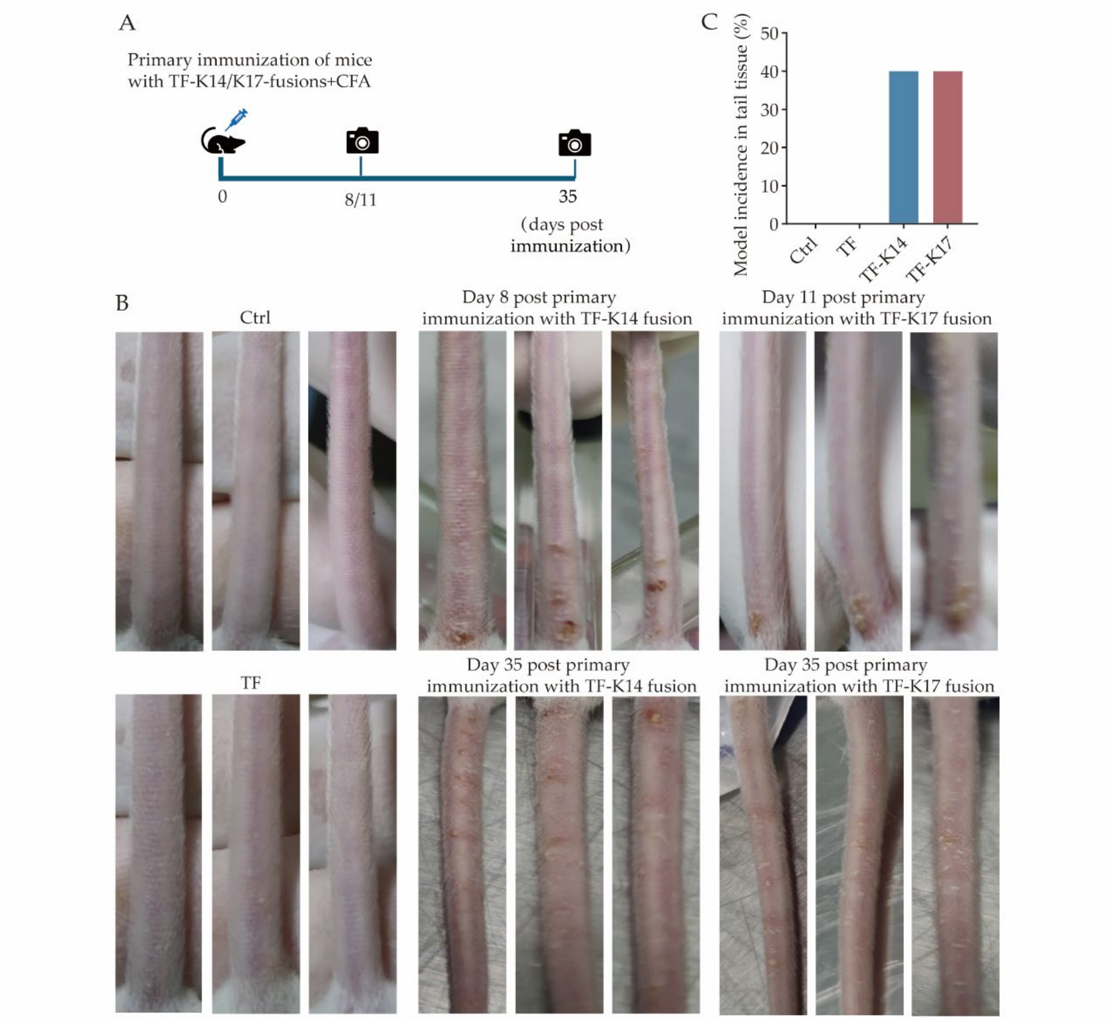

Supplementary Figure S2. Psoriasis-like symptoms in BALB/c mice immunized with TF-K14/K17. (A) Schematic diagram of the immunization strategy with TF-K14/K17 proteins in mice. (B) Symptoms in mice following immunization with TF-K14/K17 proteins. For primary immunization, 6-week-old mice were intradermally injected with 350 μg of TF (n=5), TF-K14 (n=10), or TF-K17 (n=10). Photographs of mouse tails were taken at the time points as above described in (A). Data are representative of three experiments with similar results. (C) Proportions of psoriasis mice following immunization with TF-K14/K17 proteins.

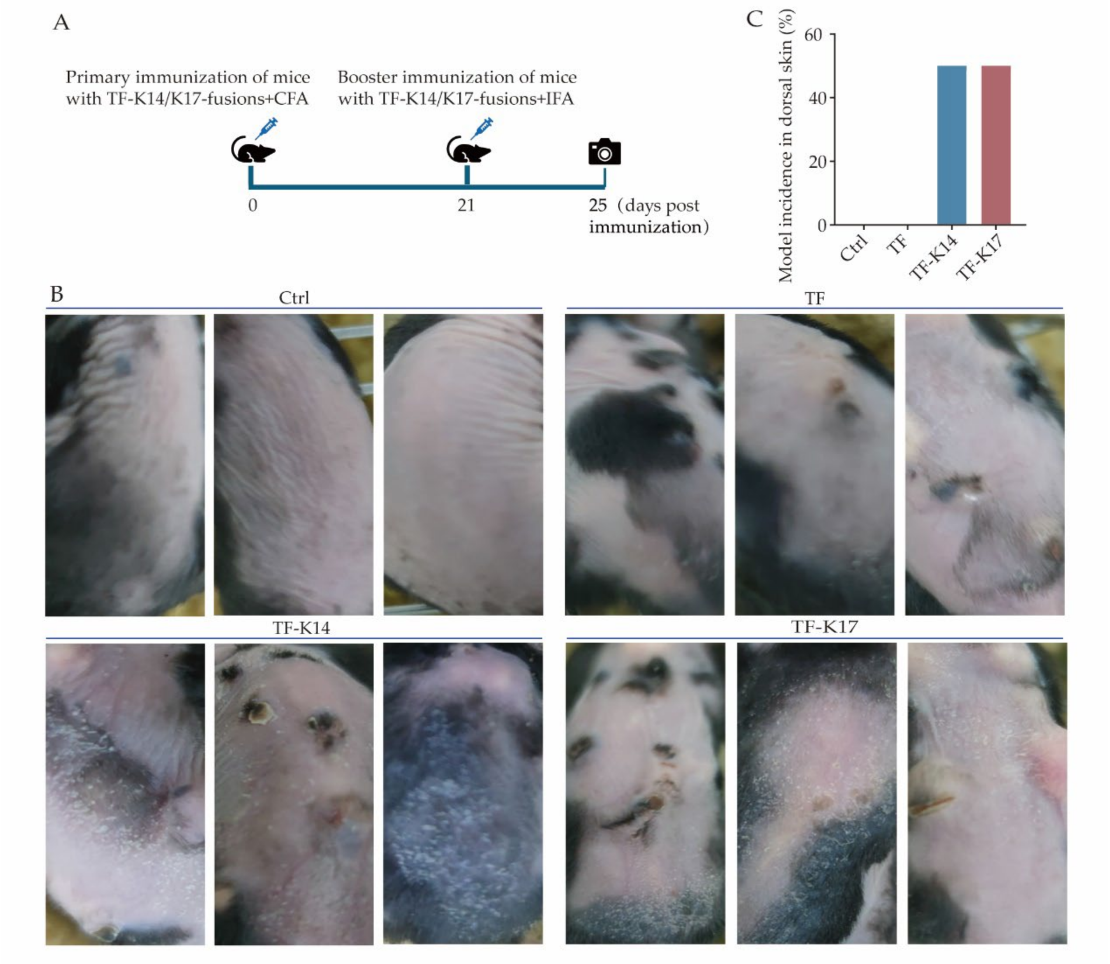

Supplementary Figure S3. Psoriasis-like symptoms in C57BL/6J mice following immunization with TF-K14/K17. (A) Schematic diagram of immunization of mouse with TF-K14/K17 proteins. (B) Psoriasis-like symptoms in mice 4 days post-booster immunization with TF-K14/K17 proteins. For primary immunization, 6-week-old mice were intradermally injected with 350 μg of TF (n=5), TF-K14 (n=10), or TF-K17 (n=10). For booster immunization, mice were subcutaneously injected with 450 μg of TF, TF-K14 or TF-K17. Photographs of mouse backs were taken at the time points as above described in (A). Data are representative of three experiments with similar results. (C) Proportions of psoriasis mice 4 days post booster immunization with TF-K14/K17 proteins.

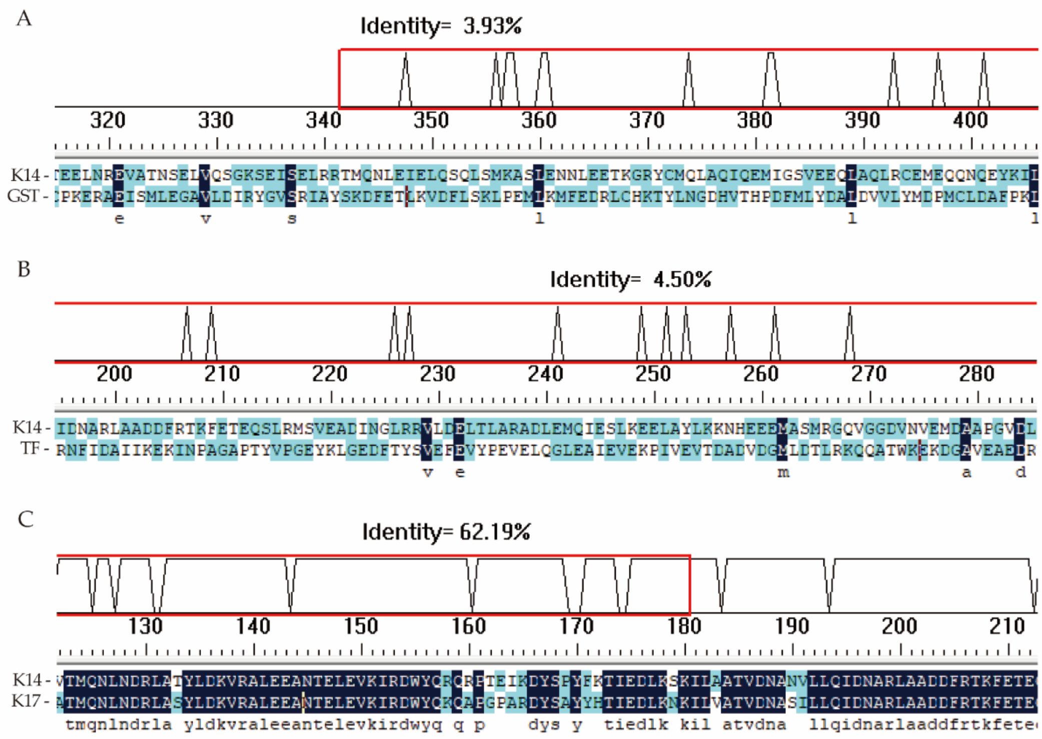

Supplementary Figure S4. Sequence homology analysis of GST, TF, K14, and K17. (A) Homology analysis of GST and murine K14 sequences using DNAMAN. (B) Homology analysis of TF and murine K14 sequences using DNAMAN. (C) Homology analysis of murine K14 and murine K17 sequences using DNAMAN.

Supplementary Table S1. Characteristics of Major Psoriasis Mouse Models.

Model Type	Incidence (Induction of Psoriasis)	Stability (Clinical Sign and Lesion Persistence and Reproducibility)	Specificity (Relevance to Clinical Features of Human Psoriasis)
Drug-Induced Model (IMQ)	Extremely high (≈ 100%). Obvious skin lesions appear in almost all mice within days of topical application.	High. Simple operation, high intra-/inter-batch consistency, however, the occurrence is acute, with rapid regression upon discontinuation, failing to reflect chronic, relapsing autoimmune disease course.	N/A. Activates TLR7 non-specifically, triggering broad-spectrum inflammation; clinical manifestations overlap with conditions such as atopic dermatitis, but it does not involve specific immune response.
Cytokine-Induced Model (IL-23, etc.)	Extremely high (up to 100%). Clinical signs and lesion induced	Moderate. Inducing acute and reversible response. Lesions regress upon	N/A. Directly targeting the IL-23/Th17 pathogenic axis, but involving exogenous

	approximately 8 days after intradermal injection.	cessation of injection, affecting only local skin without altering systemic immune status.	administration of inflammatory factors, bypassing upstream autoantigen recognition and immune initiation processes.
Xenotransplantation Model	Highly variable (depending on graft viability). Incidence influenced by patient tissue source and surgical manipulation, difficult to standardize.	Low. Limited graft survival period and high inter-batch variability due to donor tissue differences.	Very high. Retaining intact human immune and genetic background, and enabling in vivo study of human immune cells, closely resembling the real disease state in humans.
Genetically Engineered / Spontaneous Mutation Models (Antigen-Specific TCR / VEGF, etc.)	Moderate to High (Depending on the specific gene and penetrance). Transgenic models show stable disease onset once the line is established, and spontaneous mutation models exhibit fixed penetrance.	High. Clear genetic background, once successfully established and subjected to standardized stimulation. The phenotype is relatively stable, suitable for mechanistic validation.	Some limitations. Simulating specific pathological pathways driven by a single gene perturbation, failing to reflect the complex, polygenic cooperative mechanisms of disease.
Humanized Immune System Models	High variability (heavily donor-dependent).<br>Highly dependent on donor PBMC/T cell activity and individual immune status.	Low. Affected by donor variability, making it difficult to achieve highly consistent lesions across batches, A challenge to standardization and extremely high cost.	Extremely High. Recapitulates human Th1/Th17 skewing in vivo. Lesions are highly similar to that of human psoriasis.
K14/K17 Antigen-Specific mouse Model	Around 40% (variable, depending on mouse strains).<br>Induction of the disease via active immunization, a classical experimental autoimmune disease model.	Relatively high.<br>Inducing an antigen-specific immune response to autoantigen. Holding inter-batch consistency, and capable of recapitulating chronic disease features.	High. Precisely simulating the "molecular mimicry" hypothesis. Directly involving specific autoantigens, more closely recapitulating the immunological features of human psoriasis.

Note: N/A, not applicable.
