# Supplementary material for: Establishment of a Keratin 14/17-Induced Antigen-Specific Psoriasis-like Mouse Model
Source: Biology (Basel). 2026 Aug 3;15(15):1277. doi: 10.3390/biology15151277 (PMC13464592; doi:10.3390/biology15151277)
Supplement: Supplementary file 1 [file biology-15-01277-s001.zip › File S1.pdf]

Supplementary File S1: Original Images

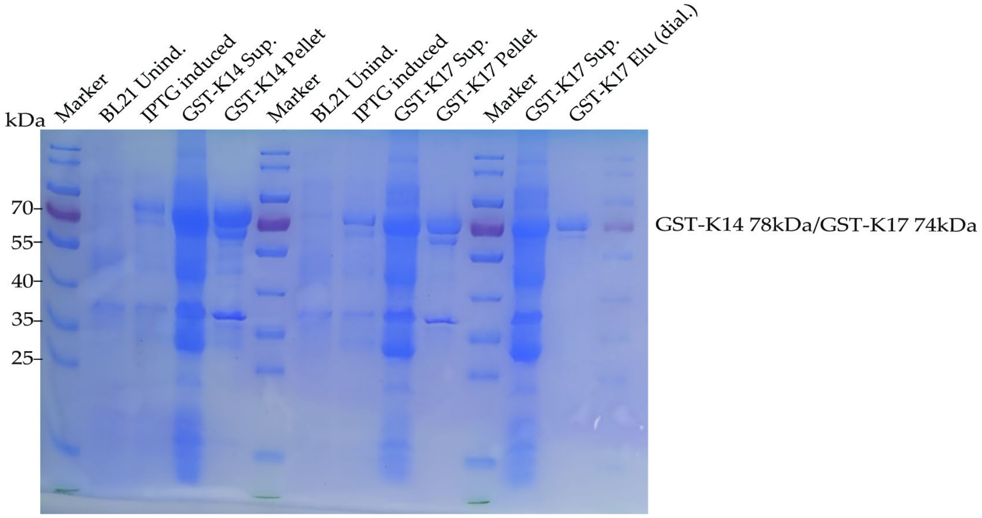

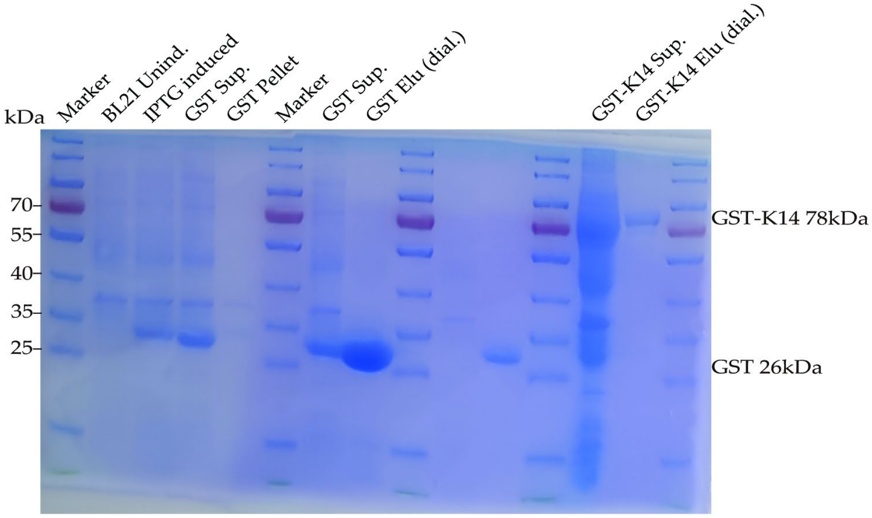

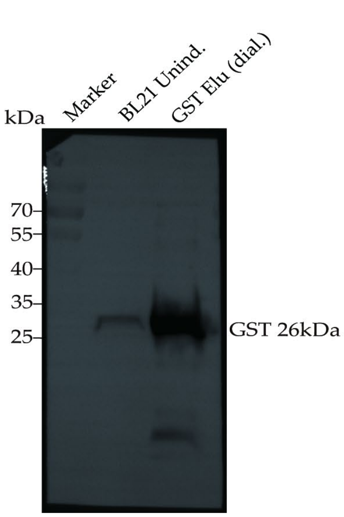

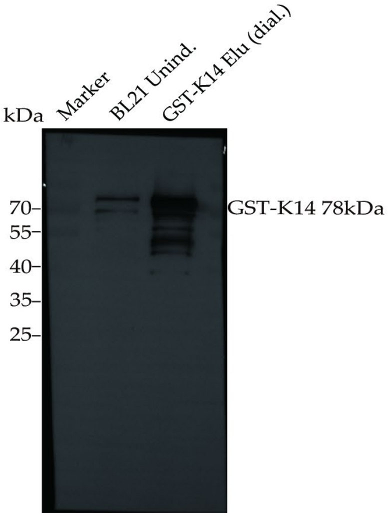

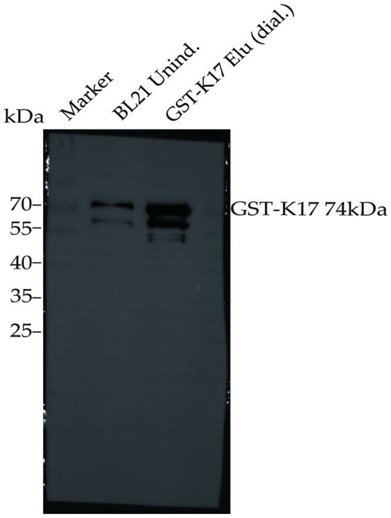

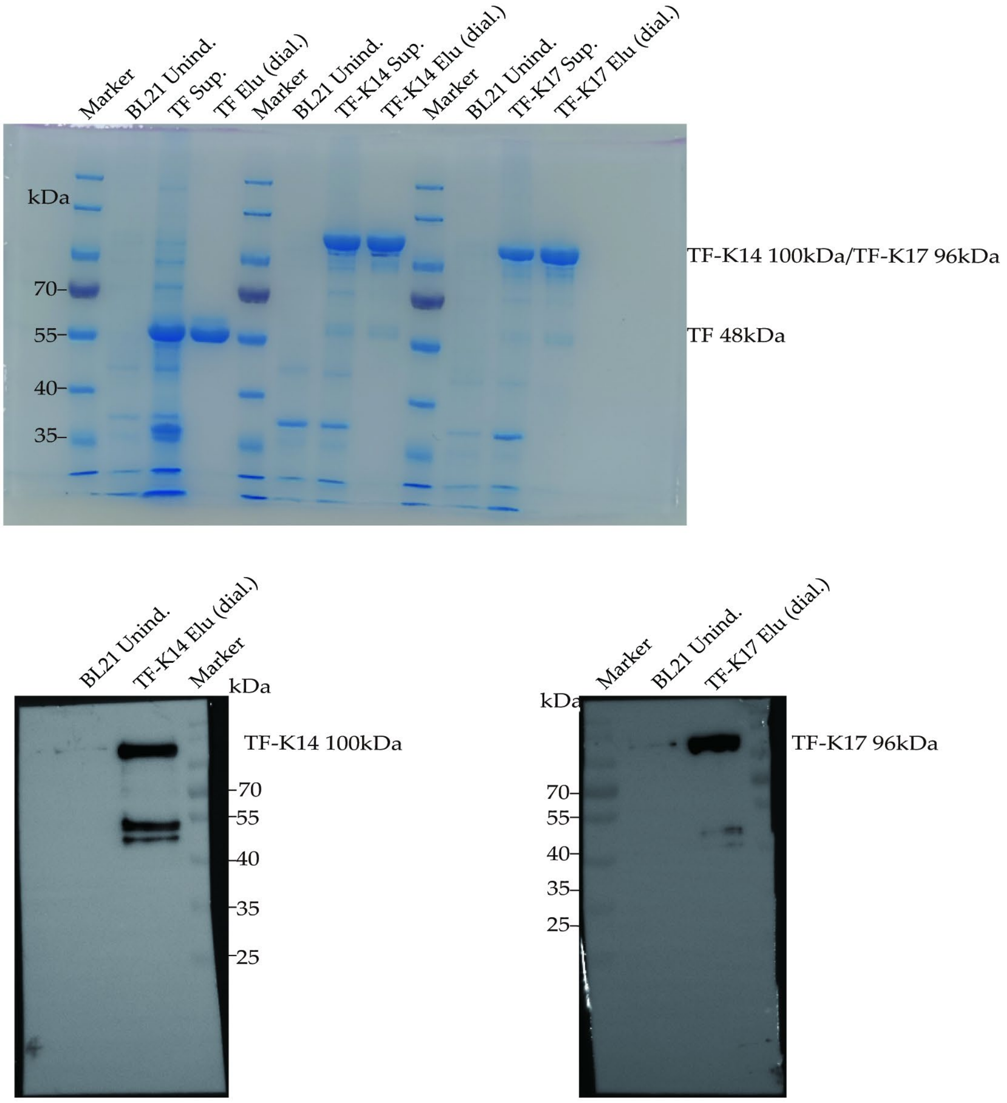

Marker
BL21 Unind.
TF Sup.
TF Elu (dial.)
Marker
BL21 Unind.
TF-K14 Sup.
TF-K14 Elu (dial.)
Marker
BL21 Unind.
TF-K17 Sup.
TF-K17 Elu (dial.)
kDa
70–
55–
40–
35–
TF-K14 100kDa/TF-K17 96kDa
TF 48kDa
BL21 Unind.
TF-K14 Elu (dial.)
Marker
kDa
TF-K14 100kDa
70
55
40
35
25
Marker
BL21 Unind.
TF-K17 Elu (dial.)
kDa
TF-K17 96kDa
70
55
40
35
25
